# Behavioral therapies for the treatment of autism spectrum disorder: A systematic review

**DOI:** 10.1016/j.clinsp.2024.100566

**Published:** 2024-12-26

**Authors:** Clara Lucato dos Santos, Indyanara Inacio Barreto, Ana Carolina Furian da Silva, Juliana Firmino Batista Soriano, Jeferson de Lima Silva Castro, Luca Schiliró Tristão, Wanderley Marques Bernardo

**Affiliations:** a Department of Evidence-Based Medicine, Faculdade de Ciências Médicas de Santos (UNILUS), Santos, SP, Brazil; b Center for Evidence-Based Medicine, Unimed Campinas, SP, Brazil; c Evidence-Based Medicine Committee at Federação das Unimeds do Estado de São Paulo (FESP), São Paulo, SP, Brazil; d Faculdade de Medicina, Universidade de São Paulo, São Paulo, SP, Brazil; e Coordinator of The Evidence-Based Medicine Department at Federação das Unimeds do Estado de São Paulo (FESP), São Paulo, SP, Brazil

**Keywords:** Autism spectrum disorder, Behavioral therapy, Healthcare setting

## Abstract

**Introduction:**

Autism Spectrum Disorder (ASD) is a neurodevelopment spectrum characterized by persistent deficits in social communication and interpersonal interaction, along with restricted and repetitive patterns of behavior, interest, or activities. The appropriate screening and diagnosis must be realized to improve the children's prognosis. Moreover, appropriate treatments are necessary to promote better social integration and development. In this scenario, this systematic review aims to evaluate the impacts of behavioral therapies applied in healthcare settings for patients with ASD.

**Methods:**

This systematic review followed the PRISMA guidelines. The databases MEDLINE, Embase, CENTRAL (Cochrane), and Lilacs were accessed, and gray and manual searches were performed. The search strategy was created with terms referring to autism and behavioral therapy. The studies were assessed qualitatively.

**Results:**

Randomized clinical trials and observational studies demonstrated improvements in cognitive and verbal components of patients who received behavioral therapies in therapeutic settings. These results indicate a positive impact of both cognitive-behavioral therapy and ESDM on the development of patients’ skills. Among the cognitive-behavioral therapies, the one based on the MASSI protocol did not impact the reduction of anxious symptoms. As for cognitive-behavioral therapy, one study demonstrated that the Behavioral Intervention for Anxiety in Children with Autism (BIACA), when compared to the Coping Cat protocol, improves cognition and reduces anxiety symptoms. Despite these results, further randomized clinical trials comparing behavioral therapies with one another are needed.

**Conclusion:**

In the context of behavioral therapy within a healthcare setting, the Early Start Denver Model (ESDM) showed improvements in the cognitive, verbal, and social aspects of the evaluated patients. Improvement in scores sometimes is achieved independently of the group and related to the time of interventions.

## Highlights

• Autism Spectrum Disorder (ASD) is a heterogeneous neurodevelopmental disorder.
• With correct screening and diagnosis, treatment must be offered to improve prognosis.
• Adequate treatment in a healthcare setting must be known.
• The Early Start Denver Model (ESDM) showed improvements in the cognitive, verbal, and social aspects.
• Time occasionally can improve some scores independently of the group.

## Introduction

Autism Spectrum Disorder (ASD) is a neurodevelopment spectrum characterized by persistent deficits in social communication and interpersonal interaction, along with restricted and repetitive patterns of behavior, interest, or activities. ASD symptoms typically emerge in childhood and can limit or impair daily functioning.^1^

With the update of the Diagnostic and Statistical Manual of Mental Disorders from DSM-IV to DSM-5, the term autism spectrum disorder was introduced, based on two main domains: social communication and Repetitive and Restricted Behaviors (RRBs). This revision aims to improve the sensitivity and specificity of diagnosis criteria.^1^^,^^2^

The International Classification of Diseases (ICD-11), implemented in 2022, aligned its terminology with DSM-5. ASD subdivisions relate to the presence or absence of intellectual disability and/or impairment in functional language. This harmonization aims to provide a more accurate and consistent approach to ASD diagnosis.^3^

In addition to the criteria established by DSM-5 and ICD-11, several screening and diagnostic tools exist for autism. Among them, the Modified Checklist for Autism in Toddlers, Revised with Follow-up (M-CHAT-R/F) stands out for screening purposes, while the Autism Diagnostic Observation Schedule (ADOS) and the Childhood Autism Rating Scale (CARS) are used for diagnosis.^4^, ^5^, ^6^, ^7^, ^8^

Interventions for children with ASD can be provided through educational practices, developmental therapies, and behavioral interventions.^9^^,^^10^ Grounded in learning principles, behavioral therapies utilize specific techniques, such as reinforcement, to encourage positive behaviors and reduce those that may have negative effects on the individual or others in their environment.^11^

Applied Behavior Analysis (ABA) is one of the behavioral approaches developed by Lovaas for children with ASD.^12^ Among the interventions derived from ABA, the Early Start Denver Model (ESDM) stands out.^9^^,^^13^ Early Intensive Behavioral Intervention (EIBI) aims to teach adaptive behaviors to children under 5 years old through an intensive treatment model.^14^ The ESDM employs teaching strategies based on ABA principles and developmentally appropriate practices in natural settings for children aged 12 to 60 months.^15^ Additionally, Cognitive Behavioral Therapy (CBT), a psychotherapeutic approach, is one of the interventions adopted for ASD, aiming to modify thought patterns and improve emotional well-being, especially in cases of associated comorbidities such as anxiety and obsessive-compulsive disorder.^16^^,^^17^

The treatment objectives for children with ASD include minimizing core deficits and associated impairments, maximizing functional independence, and preventing problematic behaviors that may interfere with functional skills. Interventions can be implemented in various environments and by different professionals, using defined curricula or guidelines.^9^

To evaluate the effectiveness and impact of treatment strategies, assessment tools are essential. The Mullen Scales of Early Learning (MSEL) evaluates early cognitive development,^18^ the Vineland Adaptive Behavior Scales (VABS) measures adaptive behavior,^19^ and the Autism Diagnostic Observation Schedule (ADOS) helps in measuring the severity of autism symptoms.^20^^,^^21^

Autism Spectrum Disorder has a profound impact on both the patient and their family, as well as the social context in which they are embedded. Therefore, specialized interventions are necessary to promote better social integration and development. In light of this scenario, this systematic review aims to evaluate the impacts of behavioral therapies applied in healthcare settings for patients with ASD.

## Methodology

This systematic review followed the principles of PRISMA (Preferred Reporting Items for Systematic Reviews and Meta-Analyses), a tool designed to guide the systematic review process.^22^ It is registered in PROSPERO: CRD42024589222.

### Search strategy

The electronic databases consulted until October 2024 were: MedLine (PubMed), Embase, Lilacs, Cochrane (CENTRAL). A manual search and grey literature search were also conducted, in addition to the Clinicaltrials.gov database. The following search strategy was utilized:

MedLine (Pubmed)/Embase/Cochrane (Central): (Autistic Disor* OR Autism OR Autistic Spectrum Disorder) AND (Applied Behavior OR ABA OR Behavior Therapy OR Cognitive Behavioral Therapy OR Early Start Denver Model OR ESDM).

Lilacs: (Autistic OR Autism) AND (Treatment OR therapy OR behavior).

### Eligibility criteria

The eligibility criteria for the studies were:

Inclusion

- Randomized Controlled Trials (RCTs) and observational studies.
- Patients with ASD.
- Behavioral intervention in a healthcare setting.
- At least part of the intervention applied by a healthcare professional.

Exclusion

- Analyses outside of the healthcare setting.
- Asperger.
- Pervasive Developmental Disorder-Not Otherwise Specified (PDD-NOS).
- Evaluation of parental outcomes.
- Interventions applied solely by parents.
- Animal studies / *in vitro* studies.

There were no restrictions regarding language or publication date.

### Data extraction

Two reviewers developed the search strategies and independently analyzed the titles and abstracts of the retrieved articles. After selecting those eligible for inclusion, the full texts were accessed. Discrepancies were discussed among the authors, with a third reviewer responsible for resolving those not agreed upon in the previous discussion.

Data extraction was performed manually by the reviewers and presented in a table. The extracted information included the surname of the first author, year of publication, study type, population characteristics, number of patients in each group, intervention, comparison, and outcomes.

### Data analysis

Each study was described individually, and its evidence was qualitatively assessed based on its outcomes. The evaluation of treatment efficacy was based on the scores applied.

If possible, the extracted data will be meta-analyzed using RevMan 5.4 software.^23^ The random-effects model will be used for results with high statistical heterogeneity (I^2^ > 50 %). For results with low heterogeneity (I^2^ < 50 %), the fixed-effects model will be employed. For categorical variables, the risk difference will be used, and for continuous variables, the mean difference will be utilized. The significance level adopted will be 0.05.

### Assessment of bias risk and evidence quality

**Fig. 1:**
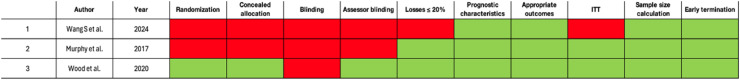
Bias assessment of randomized clinical trials.

**Fig. 2:**
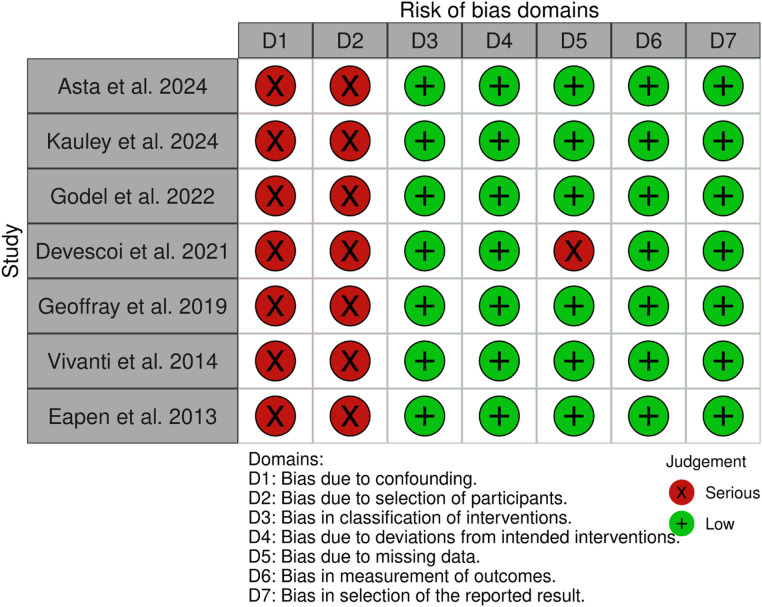
Bias assessment of non-randomized clinical trials and observational studies.

The Robins-I tool for non-randomized studies was used to assess the risk of bias in observational studies and non-randomized clinical trials. To evaluate the risk of bias in Randomized Controlled Trials (RCTs), the following criteria were employed: adequate randomization, allocation concealment, blinding of the assessor, double blinding, losses > 20 %, prognostic characteristics, intention-to-treat analysis, appropriate outcomes, sample size calculation, and adequate follow-up time. Studies were classified as high risk, some concern, or low risk (Fig. 1, Fig. 2).

## Results

**Fig. 3:**
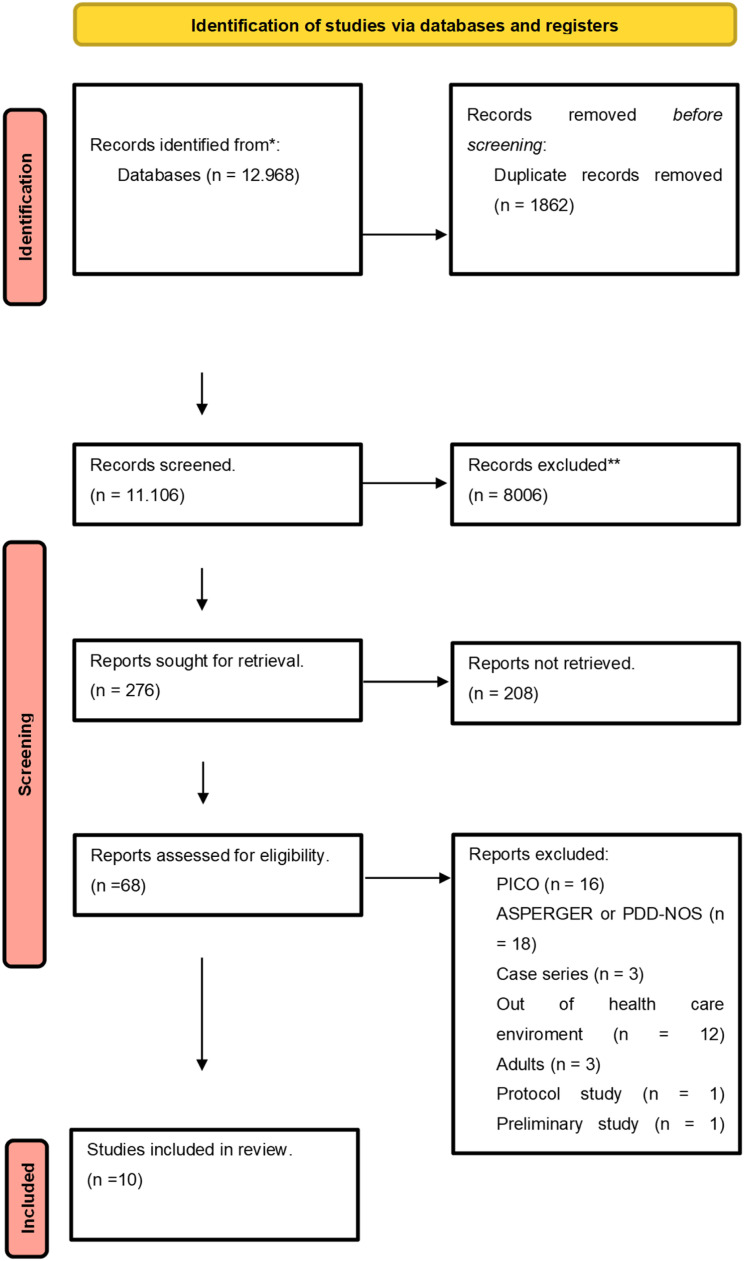
Flow diagram. *Consider, if feasible to do so, reporting the number of records identified from each database or register searched (rather than the total number across all databases/registers). **If automation tools were used, indicate how many records were excluded by a human and how many were excluded by automation tools.

The Search conducted up until October 2024 retrieved 11,106 articles after removing duplicates, of which 68 were selected for full-text review. In accordance with the eligibility criteria, 10 articles were included to support this systematic review (Fig. 3). The list of excluded articles is available in Appendix A.

### Randomized clinical trials

The randomized clinical trials evaluated the Early Start Denver Model (ESDM) and Cognitive Behavioral Therapy (CBT) as interventions. Comparisons included usual therapy and waitlist control. The characteristics of the studies and sessions can be reviewed in Table 1.

**Table 1:** Description of randomized clinical trial.

PMID	Author	Year	Study design	Intervention					Comparision				
				Intervention	n	Age	Therapy duration	Follow-up	Comparision	n	Age	Therapy duration	Follow-up
28,770,527	Murphy et al.	2017	ECR	CBT	17	14.94 (1.63) years	‒	12 weeks	MASSI	19	15.56 (1.91) years	‒	12 weeks
31,755,906	Wood et al.	2020	ECR	CBT (COPING CAT)	71	7‒13 years	60+15 min with parents on check-in (plus 2 meeting with therapist)	16 weeks	TAU	19	7‒13 years	‒	4 months
				CBT (BIACA)	77		90 min (45 min parents e 45 min individual)	16 weeks				‒	
35,697,958	Wang S et al.	2024	ECR	ESDM	30	21.14 ± 3.01 months	1 × /week 60 min (children) + parents ≥20hs/ sem	12 months	WLC	30	21.71 ± 3.10 months	‒	12 weeks

Early Start Denver Model (ESDM)

A randomized clinical trial^24^ evaluated ESDM as an intervention. One study compared it to a waitlist, two to usual therapy and other treatments, and two conducted a pre- and post-analysis. The average age of patients ranged from 21,14 to 49.6 months.

The outcomes evaluated included: GDS and PEP-3.

#### Psychoeducational profile, third edition (PEP-3)

Wang et al.^24^ demonstrated an increase in scores across all components over time at 6- and 12-months (*p* < 0.05), regardless of the allocated group. Additionally, there was an increase in scores in the following items in the ESDM group, compared to the control group (waitlist control). These items included cognitive verbal/preverbal (*p* = 0.021), social reciprocity (*p* = 0.046), and verbal behavior characteristics (*p* = 0.014). This indicates improvements in cognitive, social, and verbal components.

#### Gesell developmental scale (GDS)

In the study by Wang et al.,^24^ which compared ESDM to a waitlist control, a time effect was observed in the assessment after 12-months of follow-up, regardless of the allocated group, across all components of the GDS score (*p* < 0.05). In the 6-month assessment, a time effect independent of the group was noted for social adaptation (*p* = 0.03) and fine motor skills (*p* = 0.01). For the item speech and personal communication, the 6-month analysis from the start of interventions indicated a time effect only for speech (*p* = 0.001).

When considering the interaction between group and time, an improvement in the score was observed, indicating the benefits of ESDM for speech (*p* = 0.01) and personal communication (*p* = 0.047). However, in the 12-month analysis, the interaction between time and group showed no significant difference for any of the items (*p* > 0.05).

Cognitive Behavioral Therapy (CBT)

Two studies^25^^,^^26^ evaluated cognitive-behavioral therapy as an intervention. Murphy et al.^25^ compared it with counseling, while Wood et al.^26^ contrasted it with usual therapy. Both studies included patients diagnosed with autism spectrum disorder and anxiety.

Murphy et al.^25^ applied the Multimodal Anxiety and Social Skill Intervention for adolescents with ASD (MASSI), which addresses anxiety associated with ASD through individual therapy, group social skills training, and parental coaching. The modules include techniques such as cognitive restructuring, aimed at helping patients reformulate negative thoughts, and exposure therapy, which guides patients to gradually face anxiety-inducing situations. Additionally, the intervention acknowledges the special interest of patients with ASD, incorporating these interests into the treatment to enhance engagement during therapy.

Wood et al.^26^ implemented cognitive-behavioral therapy in two groups, with one group receiving the Coping Cat model and the Behavioral Intervention for Anxiety in Children with Autism (BIACA). The Coping Cat model focuses on recognizing anxious feelings and associated somatic reactions, identifying cognitive patterns in anxiety-provoking situations, developing strategies for managing these situations, conducting imaginary and *in vivo* exposure tasks, and reinforcing efforts through self-reward. The BIACA model follows a similar approach to Coping Cat regarding cognitive restructuring and exposure. However, it uses a modular format guided by an algorithm. Additionally, it addresses children's disruptive behaviors based on antecedent and incentive practices and teaches children necessary social engagement skills to facilitate peer-oriented exposure therapy tasks. It also treats children's special interests as assets and incorporates these interests into treatment to promote engagement.

The evaluated outcomes included: CSRs, CASI-anx, PARS, Child Behavior Checklist Scales, and SRS.

#### Clinician severity rating (CSRs)

The study by Murphy et al.^25^ compared cognitive-behavioral therapy with counseling in children with autism spectrum disorder and anxiety. The evaluation immediately after the intervention showed a difference only in separation anxiety (*p* = 0.01), while there was no difference in social anxiety (*p* = 0.31), specific phobia (*p* = 0.51), and generalized anxiety (*p* = 0.41). After the 12-week follow-up evaluation, there was no difference in any of the CSR components (*p* > 0.05)

#### Child and adolescent symptom inventory-4 asd anxiety scale (CASI-anx)

In the research conducted by Murphy et al.,^25^ no difference appeared in CASI-anx results at the immediate post-test (*p* = 0.18) and at the 12-week follow-up evaluation (*p* = 0.33). This finding indicates that there was no difference in the anxiety symptoms of the children throughout the period.

#### Pediatric anxiety rating scale (PARS)

Wood et al.^26^ demonstrated a decrease in scores among patients who received BIACA (*p* < 0.001), indicating an improvement in anxiety symptoms among the children who underwent this form of behavioral therapy. However, there was no difference in scores between those who received Coping Cat after four months (*p* > 0.05).

#### Child behavior checklist scales-2 (CBCL-2)

Wood et al.^26^ found a difference in anxiety and depression symptoms (*p* = 0.004) and the internalizing scale (*p* = 0.01) when comparing BIACA and Coping Cat, favoring BIACA and indicating an improvement in symptoms as well as externalizing behaviors and internalizing symptoms. Similarly, there was a difference when comparing BIACA and usual treatment in both items (*p* = 0.002 and *p* = 0.04, respectively), showing an improvement in scores with the intervention.

#### Social responsiveness scale-2 (SRS)

Wood et al.^26^ compared cognitive-behavioral therapy using the BIACA method with Coping Cat and usual therapy and evaluated the social communication item of the scale. A decrease in the score was observed when compared with Coping Cat (*p* = 0.04) and with usual treatment (*p* = 0.008), highlighting an improvement in communication in the patient who received the BIACA therapy.

#### Clinical improvement

Wood et al.^26^ assessed clinical improvement among the patients using the PARS severity score after treatment. In the group that received BIACA, 53 % of the children showed symptom improvement, while 37.9 % of the children who received Coping Cat and none of the 18 children who received Treatment as Usual (TAU) showed improvement (*p* < 0.001).

### Non-randomized clinical trials

Four non-randomized clinical trials were included.^27^, ^28^, ^29^, ^30^ All applied ESDM as an intervention. Eapen et al. assessed group-based ESDM, while the other authors evaluated individual sessions supplemented with parental follow-up to implement certain homework tasks. The characteristics are described in Table 2.

**Table 2:** Description of non-randomized clinical trial.

				**Intervention**					**Comparision**				
**PMID**	**Author**	**Year**	**Study design**	**Intervention**	**n**	**Age**	**Therapy duration**	**Follow-up**	**Comparision**	**n**	**Age**	**Therapy duration**	**Follow-up**
34,573,216	Devescovi et al.	2021	Clinical trial	ESDM	37	29.68 months (6.53)	‒	12 months	TAU	48	34.22 months (5.06)	‒	12 months
38,873,535	Asta et al.	2024	Clinical trial	ESDM	32	29.66 months (4.48)	6-hours per week	9 months	Before				
23,294,523	Eapen et al.	2013	Clinical trial	ESDM	26	49.6 months	15‒20 h per week group	9.72 months (2.91)	Before				
							+2:30 h - individual						
24,974,255	Vivanti et al.	2014	Clinical trial	ESDM (group)	27	18‒36 months	12‒25 h per week + parents’ feedback for 2hs in each module	12 months	Other	30	18‒36 months	‒	12 months

The evaluated outcomes were: VABS-II, ADOS-2, MSEL and SCQ.

Early Start Denver Model (ESDM)

#### Vineland adaptive behavior scales, second edition (VABS-II)

Devescoi et al.^27^ compared ESDM with usual therapy, evaluating scores at the start of treatment, after six months, and twelve months of intervention. The communication component showed an increase in score over the 12-months, irrespective of the treatment group (*p* = 0.01). In the daily living skills subscale, there was an improvement in the group that received ESDM compared to the usual therapy group (*p* = 0.01), regardless of time. The score was higher at the assessment before treatment began (*p* = 0.002) and after six months of intervention (*p* = 0.04). However, at twelve months, there was no difference between the groups (*p* = 0.05). In the socialization assessment, no difference was found between the groups during the evaluation period (*p* > 0.05). With respect to adaptive behavior, improvement was observed over time, regardless of the group (*p* = 0.09), but there was no difference between the groups or in the time-group relationship (*p* = 0.29).

Vivanti et al.^28^ assessed the VABS-ABC component of the VABS-II score. Independently of the assigned group, there was an increase in the VABS-ABC score over the 12-months (*p* = 0.01), indicating an improvement in adaptive functioning. However, there was no difference in the rate of improvement between the groups over time (*p* = 0.33), nor between the groups notwithstanding time (*p* = 0.89). This indicates that the improvement in adaptive functioning over the period occurred due to the duration of intervention and the specific treatment. In the evaluation of the VABS subscales, there was an increase in the score in the communication subscale at 12-months (*p* < 0.001), demonstrating that there was no difference in the VABS scores in the other areas.

Eapen et al.^29^ compared the VABS-II score at baseline and after an average of 9.72 (SD = 2.91) months of group ESDM. They found differences in the components of the VABS-II scale score: receptive communication (*p* = 0.005) and gross motor skills (*p* < 0.001), with increases in values. In contrast, the VABS-II Standard Domains Score revealed a difference in the motor skills component over the time interval (*p* = 0.02). However, there were no differences in the components assessing communication, socialization, daily living skills, and adaptive behavior composite. This indicates an improvement in receptive communication, gross motor skills, and motor skills among patients who received group ESDM over an average of 9.72-months.

Asta et al.^30^ conducted an assessment before and after 9-months of treatment. They found no difference in the VABS-II score evaluating daily living skills between baseline and post-intervention (*p* = 0.05).

#### ADOS-2

The ADOS-2 score in the study by Devescoi et al.^27^ was evaluated based on the ADOS Calibrated Severity Score (ADOS-CSS) through the total score and the components: social effect and restricted and repetitive behaviors. The total score on the ADOS-CSS increased over time, regardless of the group, in patients who received both ESDM and TAU (*p* < 0.001).

In the domain of assessing social effect, the study by Devescoi et al.^27^ found a significant variation over the follow-up period, independently of the assigned group (*p* < 0.001). This indicates that participants improved in the social affect domain, regardless of the type of treatment received. Additionally, there was a difference in this domain between groups, independent of time (*p* = 0.04), with a lower total score in the group that received ESDM compared to the treatment-as-usual group. This demonstrates that despite the duration of treatment, improvement primarily occurred in the intervention group. However, no difference was found when the variables of time and group were combined (*p* = 0.17), indicating that when these variables were considered together, there were no distinctions in benefits between the different treatments over time.

In the component assessing restricted and repetitive behaviors in the article by Devescoi et al.,^27^ there was a difference between the groups, regardless of the time (*p* = 0.03), indicating a variation in these behaviors among the groups. No significant difference was found when evaluating time independent of the group (*p* = 0.09) or the interaction between the group and time (*p* = 0.49). Furthermore, despite there being no significant difference in the initial characteristics of the children (*p* = 0.09), the total score on the RRB CSS was higher in the ESDM group compared to the treatment as-usual group at six and twelve months (MD = 1.24; *p* = 0.04).

Vivanti et al.^28^ assessed the ADOS-SS, and no differences were observed in the analysis over time (*p* = 0.90) or between groups over 12-months (*p* = 0.64) in the ADOS severity score. In the subscales, there was a reduction in the score assessing social effect after 12-months, independently of the group (*p* < 0.05). However, no changes were observed in the scores related to the interaction between group and time, both in the social affect domain and in the restricted and repetitive behaviors.

The clinical trial by Asta et al.,^30^ with a before-and-after analysis, showed a reduction in the total score from baseline (2.88) to after 9 months of treatment (2.71) (*p* = 0.02). In the ADOS-SA domain, there was a reduction from 14.96 at baseline to 12.96 at the 9-month assessment (*p* = 0.01), while the RRB domain showed no difference (*p* = 0.40), indicating improvement only in the social affect domain.

#### Mullen scales of early learning (MSEL)

Vivanti et al.^28^ demonstrated an increase in the MSEL Developmental Quotient (DQ), indicating an improvement in the cognitive skills in both groups (*p* < 0.001). In the analysis of the interaction between time and group, cognitive skills showed progress in both groups over time (*p* < 0.05). Additionally, there was an advancement over 12-months regardless of the group (*p* < 0.001), reinforcing cognitive development. However, no difference was observed when comparing the groups independent of time (*p* = 0.12).

Vivanti et al.,^28^ when evaluating the relationship between time and subgroups, observed differences in all components of the scale: Visual Reception (*p* < 0.001), Fine Motor (*p* = 0.01), Receptive Language (*p* < 0.001), and Expressive Language (*p* < 0.001). This indicates that the treatment over 12-months influenced the cognitive skills of the patients. In the analysis of the interaction between time and group, a variation was found only in the receptive language component (*p* < 0.05). The ESDM group showed improvement compared to the control group over the 12-month period. This suggests that the groups responded differently to the treatment during the follow-up.

In the before-and-after evaluation by Eapen et al.,^29^ there was an increase in the mean value of the following MSEL score components: Visual reception DQ (*p* = 0.001), Receptive Language DQ (*p* < 0.001), and Expressive Language DQ (*p* = 0.001). Additionally, there was an increase in the Overall MSEL DQ (*p* < 0.001), indicating an improvement in cognitive components. However, no difference was observed over the time interval for the Fine Motor DQ component (*p* = 0.28).

#### Social communication questionnaire (SCQ)

In the study by Eapen et al.,^29^ a difference was observed in the total SCQ score at the end of treatment compared to the baseline (*p* = 0.04). The initial mean SCQ score was 18.5 (SD = 7.2), and after the group ESDM intervention, it reduced to 15.7 (SD = 7.10). This indicates a decrease in characteristics associated with autism spectrum disorder, particularly in deficits related to social communication, reciprocal social interaction, and restricted and repetitive behaviors.

#### Psychoeducational profile, third edition (PEP-3)

The study by Asta al.^30^ showed an increase from 55.84 to 67.11 in the PEP-3 cognition domain score, which assesses verbal and pre-verbal behavior, between baseline and after 9-months of treatment (*p* = 0.02), indicating an improvement.

### Observational studies

Three observational studies were included in this research.^31^, ^32^, ^33^ As an intervention, the studies adopted the Early Start Denver Model. The ages of the patients ranged from 23 months to 4.7 years. The patients were predominantly male. The sessions consisted of individualized therapy with specialized therapists.

Kauley et al.^31^ divided the patients into two groups, verbal and minimally verbal, based on the ADOS-2. The detailed characteristics of the studies can be found in Table 3.

**Table 3:** Description of observational studies.

PMID	Author	Year	Study design	Intervention	n	Age	Therapy duration	Follow-up
31,596,826	Geoffray et al.	2019	Cohort	ESDM	19	34.7 (7.3) months	8.3 (1.2) hours a week	9.8 (1.2) months
38,223,372	Kauley et al.	2024	Cohort	ESDM	64	4.1 (±0.6) years	‒	15.1 (±6.3) months.
35,815,035	Godel et al.	2022	Cohort	ESDM	55	28.7 ± 5.1 months	Individual: 20h/week	2-years
							Parents: 12h/week	

The outcomes assessed included: MSEL, VABS-II, SCQ, ADOS CSS and ESDM curriculum checklist.

Early Start Denver Model (ESDM)

#### Mullen scales of early learning (MSEL)

Geoffray et al.^32^ assessed the use of ESDM after 10-months of treatment. There was an increase in the scores for the following components: overall DQ (*p* < 0.001), nonverbal DQ (*p* < 0.01), visual reception DQ (*p* < 0.01), verbal DQ (*p* < 0.01), receptive language DQ (*p* < 0.001), and expressive language DQ (*p* < 0.05). The only component that did not show a difference was fine motor DQ (*p* = 0.06).

Kauley et al.^31^ demonstrated a mean difference between the verbal and minimally verbal groups at the start of the study in the following items: visual reception (*p* < 0.012), receptive language (*p* < 0.001), and expressive language (*p* < 0.001). This indicates differences in visual perception, receptive language, and expressive language between the groups at baseline. The mean difference between the verbal and minimally verbal groups was 15.5 (95 % CI 9.6 to 21.4) in the visual component, 17.2 (95 % CI 8.1 to 26.3) in receptive language, and 13.8 (95 % CI 9.1 to 18.6) (*p* < 0.001).

#### Vineland adaptive behavior scales, second edition (VABS-II)

Geoffray et al.^32^ demonstrated a decrease in the scores related to socialization and daily living skills (*p* < 0.05), highlighting an improvement in these communication components. No differences were observed in the items: composite score, communication, and motor skills (*p* > 0.05).

The study by Godel et al.^33^ showed an increase in the total score of VABS-II adaptive behavior composite from 80.7 (SD = 12.3) to 83.3 (SD = 16.1) between 12- and 24-months, validating an improvement in the children's behavior (*p* < 0.01). No differences were observed in the components: socialization, daily living skills, and motor skills. In the communication component, there was an increase in the score from baseline to 12-months (*p* < 0.01), from baseline to 24-months (*p* < 0.01), and from 12- to 24-months (*p* < 0.01), indicating progress in the children's communication over time.

Kauley et al.^31^ evaluated the maladaptive behavior score component of the VABS. No differences were found between the verbal and minimally verbal groups, both at baseline (*p* = 0.42) and at the end of the follow-up (*p* = 0.18). This indicates that there was no difference between the groups or over time in this behavior assessment.

#### Autism diagnostic observation schedule calibrated severity score (ADOS CSS)

Godel et al.^33^ showed a decrease in the total ADOS CSS score between baseline and 12-months (*p* < 0.001) and between baseline and 24-months (*p* < 0.001), indicating an enhancement in the children's behavior patterns. However, no difference was found in the score between 12- and 24-months (*p* = 1.00). In the component specifically assessing social effect, there was a decrease in the score between baseline and both 12-months and 24-months (*p* < 0.001), indicating an improvement in the children's social affect expression. The component assessing restricted and repetitive behaviors showed a decrease in the score between baseline and 12-months, indicating progress in restrictive and repetitive symptoms over the period (*p* < 0.001).

### ESDM curriculum checklist

Kauley et al.^31^ demonstrated a difference in the ESDM receptive communication item between the verbal and minimally verbal groups at the end of the follow-up, with values of 27.8 (SD = 8.6) and 19.3 (SD = 6.6), respectively (*p* < 0.01), indicating progress in the ability to understand a message, sentence, or command issued by another person. After the follow-up, an improvement was observed between the groups in the ESDM expressive communication, suggesting an enhancement in the ability to communicate with others (*p* < 0.001), and in the ESDM social skills, reflecting progress in the ability to interact with others (*p* = 0.001).

### Bias analysis

Randomized clinical trials

The risk of bias was high in two randomized clinical trials, with inadequate randomization, blinding allocation, and blinding between the groups. The clinical trial by Wood et al. has a low risk of bias.

Non-randomized clinical trials and observational studies

The risk of bias in non-randomized clinical trials and observational studies was considered moderate, primarily due to confounding factors and selection bias.

## Discussion

Randomized clinical trials and observational studies demonstrated improvements in the cognitive and verbal components of patients who received behavioral therapies in therapeutic settings. These results indicate a positive impact of both cognitive-behavioral therapy and ESDM on the development of patients’ skills. Among the cognitive-behavioral therapies, the one based on the MASSI protocol did not impact the reduction of anxious symptoms. As for cognitive-behavioral therapy, the Behavioral Intervention for Anxiety in Children with Autism (BIACA), when compared to the Coping Cat protocol, improves cognitive symptoms and reduces anxiety symptoms, making it a viable alternative for individuals with autism spectrum disorder and anxiety-related comorbidities, however, only one study evaluated these interventions.

However, it is important to highlight that the improvement observed in some scales, such as the VABS-II and ADOS CSS scales for the ESDM intervention, in the article by Devescoi et al.,^27^ was more related to the duration of follow-up than directly to the intervention itself. This suggests that, regardless of the allocation group, simply participating in some type of intervention over time may lead to improvements in cognitive and behavioral symptoms. These findings emphasize the importance of consistent adherence and adequate follow-up of therapies to maximize their benefits.

Furthermore, although behavioral therapies are conducted by trained professionals in healthcare settings, there is a clear need for therapeutic principles to be applied in the home environment as well. To achieve this, many interventions actively involve parents and caregivers, providing feedback on the treatment and training them to correctly apply therapeutic techniques in the child's routine. However, it should be considered that either the lack of application or the incorrect application of these techniques in the home environment may compromise the development of cognitive and behavioral skills.

The increase in ASD diagnoses drives the search for new therapeutic approaches. Providing effective treatments based on robust scientific evidence is an ethical responsibility for healthcare professionals. It is up to these professionals to critically assess the available evidence and make informed decisions to optimize therapeutic outcomes.

Implementing appropriate therapeutic interventions for children with ASD can significantly enhance their social, behavioral, and cognitive skills, directly impacting the patient's quality of life. Furthermore, families also benefit by gaining a better understanding of how to handle everyday situations and by applying more effective support strategies in the home environment.

ASD is often associated with other conditions, such as anxiety, depression, and obsessive-compulsive disorder. In this context, behavioral therapies not only promote improvements in cognitive and behavioral development but can also alleviate symptoms of associated disorders. However, it is important to consider that these associated disorders may pose additional barriers to the development of cognitive, behavioral, and verbal skills in children.

It is also worth noting that many patients assessed had already received prior interventions, both medicinal and non-medicinal, with some continuing to use controlled medications during the study.

This systematic review has some limitations. The included studies featured interventions conducted in healthcare settings, thus primarily excluding studies based on naturalistic practices, which are often applied in school, home, or routine contexts for the child. Additionally, studies in which interventions were administered exclusively by caregivers, parents, or teachers were excluded, which significantly reduced the number of included articles.

Additionally, the transition from the DSM-IV to the DSM-5 impacted the classification of the disorder. Previously, classification included childhood disintegrative disorder, pervasive developmental disorder not otherwise specified, autistic disorder, Asperger's disorder, and global developmental disorder, based on social communication, restricted and repetitive behaviors, and communication deficits. With the change, the DSM-5 defines Autism Spectrum Disorder, which can be classified from mild to severe based on social communication and restrictive repetitive behaviors. Under this new classification, currently in use, it is not possible to ensure that all patients previously classified with Asperger's disorder or global developmental disorder, for example, would be included in the ASD diagnosis according to the DSM-5 criteria and, therefore, were not included.

The inability to perform a quantitative assessment through meta-analysis is a limitation of this study, as the interventions applied, and outcomes assessed vary considerably across the included studies. This diversity complicates the direct comparison of results and the quantitative synthesis of evidence, especially considering that some outcomes are qualitative and thus not measurable.

Finally, no articles were found that directly compare behavioral therapies with each other. Randomized clinical trials comparing behavioral therapies across different age groups of children diagnosed with ASD, without prior treatment, and with adequate follow-up, are needed. Given the importance of therapeutic approaches for children, new studies with methodological rigor are essential, aiming to provide effective and high-quality treatment to improve the prognosis of patients with ASD.

## Conclusion

In the context of behavioral therapy within a healthcare setting, the Early Start Denver Model (ESDM) showed improvements in the cognitive, verbal, and social aspects of the evaluated patients, demonstrating its positive impact on overall development. Improvement in some scores sometimes is achieved independently of the group and related to the time of interventions. Despite these results, further studies comparing behavioral therapies with one another are needed.

## Conflicts of interest

The authors declare no conflicts of interest.

## Appendix Supplementary materials

Image, application 1
