# Supplementary material for: Behavioral therapies for the treatment of autism spectrum disorder: A systematic review
Source: Clinics (Sao Paulo). 2024 Dec 26;80:100566. doi: 10.1016/j.clinsp.2024.100566 (PMC11732605; doi:10.1016/j.clinsp.2024.100566)
Supplement: Supplementary file 1 [file mmc1.docx]

**CLINICS-D-24-01222_Supplementary Material**

**Appendix A** Exclusion reason.

| **Excluded** | | | | |
| --- | --- | --- | --- | --- |
|  | **PMID** | **Author** | **Year** | **Reason** |
| **1** | 36847894 | Fuselier et al. | 2024 | PICO |
| **2** | 33705167 | Wood et al. | 2021 | PICO |
| **3** | 36053934 | Clifford P et al. | 2022 | PICO |
| **4** | 32219638 | Kilburn et al. | 2020 | PICO |
| **5** | 30957534 | Klebanoff et al. | 2019 | PICO |
| **6** | 27020055 | Vivanti et al. | 2016 | PICO |
| **7** | 27040557 | Maddox et al. | 2017 | PICO |
| **8** | 38009186 | Schwartzman et al | 2024 | PICO |
| **9** | 36593287 | Mounzer et al. | 2023 | PICO |
| **10** | 35624439 | Choi et al. | 2022 | PICO |
| **11** | 32642958 | Dimian et al | 2021 | PICO |
| **12** | 32026260 | McBride et al. | 2020 | PICO |
| **13** | 31107360 | Holzinger et al. | 2019 | PICO |
| **14** | 29110508 | Colombi et al. | 2016 | PICO |
| **15** | 25526831 | Wood et al. | 2015 | Asperger |
| **16** | 19562475 | Wood et al. | 2009 | Asperger + PDD NOS |
| **17** | 32404180 | Solish et al. | 2020 | Asperger |
| **18** | 33826038 | Storch et al. | 2022 | Asperger + PDD NOS |
| **19** | 30293128 | Vause et al. | 2020 | Asperger + PDD NOS |
| **20** | 26088663 | Estes et al. | 2015 | Asperger + PDD NOS |
| **21** | 25424398 | Storch et al. | 2015 | Asperger + PDD NOS |
| **22** | 24671750 | Wood et al. | 2014 | PDD-NOS |
| **23** | 23357440 | Storch et al. | 2013 | Asperger |
| **24** | 22435114 | Reaven et al. | 2012 | Asperger |
| **25** | 21660428 | Sung et al. | 2011 | Asperger + PDD NOS |
| **26** | 19948568 | Dawson et al. | 2010 | Asperger + PDD NOS |
| **27** | 19309326 | Wood et al. | 2009 | Asperger + PDD NOS |
| **28** | 38618362 | Peterson et al | 2024 | Asperger + PDD NOS |
| **29** | 34342287 | Choi et al. | 2022 | Asperger |
| **30** | 29726269 | Rivard et al. | 2018 | Asperger + PDD NOS |
| **31** | 30009626 | Waters et al. | 2018 | Asperger + PDD NOS |
| **32** | 20508979 | Drahota et al. | 2010 | Asperger |
| **33** | 15358873 | bernard-opitz et al. | 2004 | Case series |
| **34** | 35309698 | Tupou et al. | 20222 | Case series |
| **35** | 21457605 | Scarpa et al. | 2011 | Case series |
| **36** | 24847474 | Fulton et al. | 2014 | Ambiente |
| **37** | 32744133 | Mazza et al. | 2020 | School |
| **38** | 17438342 | Eikeseth et al. | 2007 | Not in healthcare setting |
| **39** | 37171764 | Bent et al | 2024 | Not in healthcare setting |
| **40** | 16477514 | Elvevdik et al. | 2006 | Not in healthcare setting |
| **41** | 27807755 | Leaf et al. | 2017 | Not in healthcare setting |
| **42** | 17963434 | Remington et al. | 2007 | Not in healthcare setting |
| **43** | 30768394 | rogers et al | 2019 | Not in healthcare setting |
| **44** | 30768394 | rogers et al | 2019 | Not in healthcare setting |
| **45** | 30632299 | Smith et al. | 2018 | Not in healthcare setting |
| **46** | 20627451 | Peters-scheffer et al. | 2010 | Not in healthcare setting |
| **47** | 35182258 | Vinen et al. | 2023 | Not in healthcare setting |
| **48** | 36523754 | Zwilling M et al. | 2022 | Adults |
| **49** | 38435191 | Peterson et al | 2024 | Adults |
| **50** | 38435164 | Peterson et al | 2024 | Adults |
| **51** | 33424240 | Tateno et al. | 2021 | Preliminary |
| **52** | 18363003 | Ooi et al. | 2008 | Pilot |
| **53** | 28348195 | Touzet et al. | 2017 | Pilot |
| **54** | 30203308 | rogers et al | 2019 | Parents evaluation |
| **55** | 23021480 | Rogers et al | 2012 | Parents evaluation |
| **56** | 21586502 | Kovshoff et al. | 2011 | Parents intervention |
| **57** | 31787421 | Kim et al. | 2019 | PICO |
| **58** | 29609630 | Kilburn et al. | 2018 | Protocol |
| **59** | 29687457 | Weiss et al | 2019 | PICO |

**Appendix B** Abbreviations.

| **ASD** | Autism Spectrum Disorder |
| --- | --- |
| **RRBs** | Repetitive and restricted behaviors |
| **ICD-11** | The International Classification of Diseases |
| **M-CHAT-R/F** | Modified Checklist for Autism in Toddlers, Revised with Follow-up |
| **ADOS** | Autism Diagnostic Observation Schedule |
| **CARS** | Childhood Autism Rating Scale |
| **ABA** | Applied Behavior Analysis |
| **ESDM** | Early Start Denver Model |
| **EIBI** | Early Intensive Behavioral Intervention |
| **CBT** | Cognitive Behavioral Therapy |
| **MSEL** | Mullen Scales of Early Learning |
| **VABS** | Vineland Adaptive Behavior Scales |
| **PEP-3** | Psychoeducational Profile, Third Edition |
| **GDS** | Gesell developmental scale |
| **MASSI** | Multimodal Anxiety and Social Skill Intervention for adolescents with ASD |
| **BIACA** | Behavioral Intervention for Anxiety in Children with Autism |
| **CSRs** | Clinician Severity Rating |
| **CASI-anx** | Child and Adolescent Symptom Inventory-4 ASD Anxiety Scale |
| **PARS** | Pediatric Anxiety Rating Scale |
| **SRS** | Social Responsiveness Scale-2 |
| **VABS-II** | Vineland Adaptive Behavior Scales, Second Edition |
| **VABS ABC** | Vineland Adaptive Behavior Scales, Adaptive Behavior Composite |
| **ADOS CSS** | ADOS Calibrated Severity Score |
| **ADOS-SS** | ADOS severity score |
| **ADOS-SA** | ADOS Social Affect |

**Appendix C** Tests assessing domains.

| **Test** | **Domais** |
| --- | --- |
| GDS | - Motor development |
|  | - Adaptive behavior |
|  | - Language development |
|  | - Social development |
|  | - Cognitive development |
| MSEL | - Cognition development |
|  | - Motor skills |
| VABS-II | - Adaptive behavior |
| ADOS-2 | - Communication skills |
|  | - Social Interaction |
|  | - Restricted and repetitive behavior |
| PEP-3 | `- Cognitive verbal/preverbal scale |
| CSRs | - Overall symptom severity |
|  | - Functional impact |
|  | - Behavior |
| PARS | - Frequency of anxiety sympyoms |
|  | - Severity of symptoms |
|  | - Impairment on daily life |
|  | - Physical symptom |
|  | - Avoidance behavior |
| CBCL-2 | - Internalizing problems |
|  | - Externalizing problems |
|  | - Social problems |
|  | - Thought |
|  | - Attention |
| SRS | - Social awareness |
|  | - Social cognition |
|  | - Social communication |
|  | - Social motivation |
|  | - Restricted interests and repetitive behavior |
| SCQ | - Social interaction |
|  | - Communication skills |
|  | - Repetitive and Restrictive behavior |
|  | - Adaptive functioning |
| ESDM Curriculum check | - Receptive communication |
|  | - Expressive communication |
|  | - Social skills |
|  | - Cognitive skills |
|  | - Fine and gross motor skills |
|  | - Adaptive behavior |
